# CO_2_ or air cholangiography reduces the risk of post-ERCP cholangitis in patients with Bismuth type IV hilar biliary obstruction

**DOI:** 10.1186/s12876-020-01341-9

**Published:** 2020-06-15

**Authors:** Wen-hui Zhang, Peng-peng Ding, Lei Liu, Yan-ling Wang, Wen-hui Lai, Jing-jing Han, Jun Han, Han-wei Li

**Affiliations:** Diagnosis and Treatment Center of Liver Cirrhosis, 302 Hospital of PLA, Beijing, China

**Keywords:** CO_2_ cholangiography, Air cholangiography, Contrast cholangiography, Cholangitis, Hilar biliary obstruction, ERCP

## Abstract

**Background:**

Endoscopic biliary stenting by endoscopic retrograde cholangiopancreatography (ERCP) is the most common form of palliation for malignant hilar obstruction. However, ERCP in such cases is associated with a risk of cholangitis. The incidence of post-ERCP cholangitis is particularly high in Bismuth type IV hilar obstruction, and this risk is further increased when the contrast injected for cholangiography is not drained. The present study aims to compare the incidence of cholangitis associated with the use of a contrast agent, air and CO_2_ for cholangiography in type IV hilar biliary lesions.

**Methods:**

The clinical data of consecutive 70 patients with type IV hilar obstruction, who underwent ERCP from October 2013 to November 2017, were retrospectively analyzed. These patients were divided into three groups based on the agent used for cholangiography: group A, contrast (*n* = 22); group B, air (*n* = 18); group C, CO_2_ (*n* = 30). These three methods of cholangiography were chronologically separated. Prior to the ERCP, MRCP was obtained from all patients to guide the endoscopic intervention.

**Results:**

At baseline, there was no significant difference in terms of the patient’s age, gender, symptoms and liver function tests among the three groups (*P >* 0.05). The complication rates were significantly higher in group A than in groups B and C (63.6% vs. 26.7 and 27.8%, *P <* 0.05). The incidence of post-ERCP cholangitis was significantly higher in group A (*P <* 0.05), while the incidence of post-ERCP pancreatitis and bleeding were similar in the three groups. After the ERCP, the mean hospital stay was shorter in groups B and C, when compared to group A (*P <* 0.05). However, there was no significant difference in the 30-day mortality rate among the three groups (*P >* 0.05). Furthermore, there was no significant difference between groups B and C in terms of primary end points.

**Conclusion:**

CO_2_ or air cholangiography during ERCP for type IV hilar obstruction is associated with reduced risk of post-ERCP cholangitis, when compared to conventional contrast agents.

## Background

Most patients with malignant hilar biliary obstruction have an unresectable disease. These patients carry a poor prognosis, and endoscopic retrograde cholangiopancreatography (ERCP) is the standard procedure for the palliation of obstructive jaundice [1–3]. Despite the use of antibiotic prophylaxis, the risk of post-ERCP cholangitis has become particularly high in patients with Bismuth type IV hilar obstruction: strictures that involve the common hepatic duct and bilateral proximal hepatic ducts, up to the segmental bile ducts [2]. The development of cholangitis leads to prolonged hospital stay, increased mortality, repeated endoscopic interventions and percutaneous drainage [4–6]. The risk of cholangitis is further increased when the injected contrast cannot be drained during ERCP. Recently, some studies have investigated the safety of air cholangiography, and suggested that this can reduce the risk of post-ERCP cholangitis, when compared to contrast injection [7–9]. However, air embolism has been reported during ERCP, and is a known complication when air is utilized for insufflation during endoscopy. Furthermore, the mortality for air embolism can reach up to 40% [10–13]. CO_2_ has been widely used in gastrointestinal endoscopy for insufflations [14–18]. CO_2_ cholangiography is feasible and cheap. Furthermore, CO_2_ is absorbed by the gut and tissue at approximately 160 times faster than nitrogen, which is the major component of air. In addition, CO_2_ embolism has been commonly reported in laparoscopic procedures, such as laparoscopic radical prostatectomy. However, there is no reported cardiorespiratory instability associated with CO_2_ embolism [19]. To the best of our knowledge, CO_2_ embolism-related mortality is extremely rare during ERCP. One fatality was recently reported after direct peroral cholangioscopy, and this might be due to the pre-existing biliovenous shunt [20]. The aim of the present study was to compare the risk of cholangitis associated with contrast agent, air and CO_2_ injection in patients with type IV hilar lesions during ERCP.

## Methods

Between October 2013 and November 2017, all patients with type IV hilar obstruction, who underwent ERCP at the Department of Diagnosis and Treatment Center of Liver Cirrhosis, Beijing 302 Hospital, China, were included in the present study. The present study was approved by the Institutional Review Board of Beijing 302 Hospital. Prior to ERCP, MRCP was obtained from all patients to determine the ductal anatomy, and guide the endoscopic intervention. The type of hilar stricture was classified based on the Bismuth classification [2, 3]. None of these patients received prior percutaneous drainage. A written informed consent was obtained from all patients. These patients were divided into three groups based on the agent used for the cholangiography: group A, contrast (*n* = 22); group B, air (*n* = 18); group C, CO_2_ (*n* = 30). These three methods of cholangiography were chronologically separated. For all patients, biliary stenting or nasobiliary drainage was performed to drain the intrahepatic biliary system during the ERCP. The primary study outcome was post-ERCP cholangitis within four weeks after the procedure. The secondary outcomes were other ERCP complications, such as pancreatitis, bleeding, perforation and cholecystitis, hospital stay after the procedure, total serum bilirubin levels at one week after the procedure, and mortality at one month.

### ERCP procedure

All patients underwent ERCP under monitored sedation using propofol. Prophylactic broad-spectrum antibiotic was initiated at the day before the ERCP, and was continued for three days, post-procedure. With the help of the MRCP, the target intrahepatic bile duct was selected. After deep biliary cannulation, one or two guidewires (JagWire, Boston Scientific, Natick, MA, USA) were inserted through the hilar strictures, into the significantly dilated target intrahepatic duct (IHD). Then, the bile was aspirated using a sphincterotome (Autotome, Boston Scientific) once the intrahepatic duct was cannulated for confirmation, in order to decrease the intra-ductal pressure. During the initial study period, from October 2013 to February 2015, the investigators used conventional contrast (iopamidol; Bracco Sine Pharmaceutical Corp., Shanghai, China) for the cholangiography. This practice was changed to air (10–20 mL) from May 2015 to June 2016, and finally changed to CO_2_ (10–20 mL) during the last part of the study period (July 2016 to November 2017, Fig. 1).

**Fig. 1:**
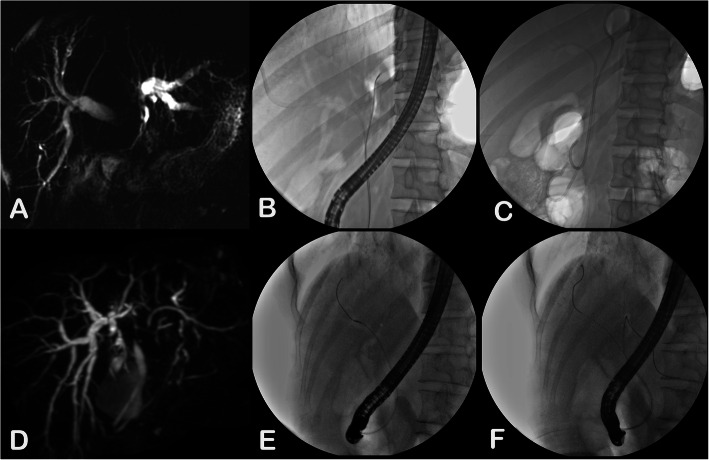
The fluoroscopic images show the air and CO_2_ cholangiograms performed during the ERCP. The MRCP image of a patient shows the type IV hilar obstruction (**a**). The air cholangiogram of the same patient (**b**)**,** followed by bilateral stenting (**c**). The MRCP image of another patient with type IV hilar obstruction (**d**). The CO_2_ cholangiogram of the same patient shows the selective wire cannulation of the right (**e**) and left (**f**) intrahepatic biliary system

An Olympus air pump with carbon dioxide gas was used. First, the pause button was pressed. Then, a 20-mL syringe was placed at the outlet, and the start button was pressed to fulfill the syringe through the positive pressure. There was no need to pump back. The visualized ductal system was compared with the MRCP images. If the selected IHD was confirmed to be the target duct suggested by the MRCP, after aspiration of the injected agents, one or two 7 French × 7–9 cm plastic stents (double-pigtails, Boston Scientific) were placed. If the cannulated duct was not considered the target duct, a 7 French nasobiliary tube (Microinvasive, Nanjing, China) was placed, in order to determine whether biliary drainage would occur. The drainage effect was evaluated for seven days. If the temporary drainage was effective, a plastic stent was introduced. Otherwise, percutaneous drainage was performed, and the nasobiliary drain was removed.

Technical success was defined by fluorography and visualization of the bile flow through the stent. Clinically successful drainage was defined as a decrease in total bilirubin level to < 2 mg/dL (the normal total bilirubin level is < 1 mg/dL) within four weeks, without moderate/severe postprocedural cholangitis, and the need for additional endoscopic or percutaneous intervention [1]. After the ERCP, the complications were documented and managed. Cholangitis was defined as a new onset of fever (temperature of ≥38 °C) without any source of infection outside the biliary system, which persists for > 24 h after the procedure. Pancreatitis was defined as a new onset of abdominal pain with a serum amylase level of three times the normal level after ERCP, requiring new admission or prolongation of the planned admission. A hemoglobin level drop of > 3 g/dL, with/without transfusion, indicated bleeding. Perforation was defined as any leak of fluid or contrast that needed further treatment, including surgery or percutaneous drainage [21]. These patients were re-evaluated in clinic at one week and one month after the procedure.

### Statistical analysis

These patients were divided into three groups based on the agent used for cholangiography for the statistical analysis. The categorical variables were expressed in numbers and percentages, and analyzed using chi-square test (correction α’ = α/[k(k-1)/2] when comparing pairs within a group). Normality tests were conducted using the Shapiro–Wilk test. Continuous variables were reported as mean ± standard deviation (SD) for normally distributed and homogeneous variables, or median and interquartile range [IQR] for non-normally distributed or non-homogeneous variables. Continuous variables were compared using the SNK-q test (between-group comparisons), three-group comparisons for normally distributed and homogeneous variables were performed using ANOVA, and Wilcoxon rank sum test (between-group comparisons, corrected α’ = α/[k(k-1)/2]) or Kruskal–Wallis was used for non-normally distributed or non-homogeneous variables. The statistical analysis was performed using the SAS software version 9.4. The statistical significance was defined as a two-sided *P* < 0.05.

## Results

A total of 70 patients with Bismuth type IV hilar biliary obstruction, who underwent ERCP at our center between October 2013 and November 2017, were included in the present study. The baseline clinical and biochemical characteristics of these patients are presented in Table 1. There was no significant difference in age, gender, symptoms and liver function tests among these three groups (Table 1, *P >* 0.05). All procedures were performed by an experienced senior endoscopist (WHZ).

**Table 1:** Baseline characteristics of the 70 patients with Bismuth IV hilar obstruction

	Contrast agent	Air	CO_2_	*P-*values
Study time frames	October 2013 – February 2015	May 2015–June 2016	July 2016 – November 2017	
Patients	22	18	30	
Age	57.74 ± 9.34	58.32 ± 13.66	58.64 ± 11.52	0.523
Male-to-Female ratio	16:6	14:4	19:11	0.60
Symptoms				
Jaundice	22 (100%)	18 (100%)	30 (100%)	> 0.99
Abdominal pain	6 (27.3%)	3 (16.7%)	4 (13.3%)	0.46
Fever	2 (9.1%)	4 (22.2%)	7 (13.3%)	0.391
Pruritus	6 (27.3%)	3 (16. 7%)	5 (16.7%)	0.647
WBC (×10^9^/L)	7.15 ± 2.65	8.70 ± 3.82	7.17 ± 3.30	0.131
Liver function tests				
Serum bilirubin (umol/L)	320.39 ± 100.86	264.23 ± 82.92	252.89 ± 74.94	0.976
ALP (median, [Q1, Q3])	450.21 (110.35, 609.14)	413.97 (125.39, 598.16)	501.47 (150.46, 622.16)	0.108
γ-GT (median, [Q1, Q3])	350.69 (169.83, 626.72)	410.34 (209.83, 598.14)	408.91 (217.38, 663.27)	0.195

Compared to the contrast injection, the image quality of air and CO_2_ cholangiograms was considered to be feasible for diagnostic and therapeutic ERCP. Technical success, including stenting and nasobiliary tube insertion, was achieved for all patients. Successful clinical drainage with relief or improvement in obstructive symptoms was observed in 74.3% of these patients (52/70). There was no significant difference in the number of stents and nasobiliary drainage tubes used in the three groups (*P >* 0.05, Table 2). The incidence of post-ERCP complications were significantly higher in group A (63.6%), when compared to group B (27.8%) and group C (26.6%) (*P* = 0.018, Table 2). The incidence of cholangitis was significantly lower in group B and group C, when compared to group A (16.7 and 10.0% vs. 50.0% respectively, *P* = 0.004). Between groups B and C, there was no statistical difference in the incidence of cholangitis (16.7% vs. 10%, *P* = 0.658; Table 2). The incidence of post-ERCP pancreatitis and bleeding were similar among the groups. No other major ERCP complications were observed among the study patients. The post-ERCP hospital stay was significantly shorter in groups B and C, when compared to group A (Table 2, *P* = 0.04), while there was no significant difference in hospital stay between groups B and C (Table 2, *P* = 0.970). During the follow-up, a repeat ERCP was performed for three patients in group C to exchange the nasobiliary tubes with biliary stents, while merely one patient needed a repeat ERCP in groups A and B (*P* = 0.854). No significant difference was observed in the 30-day mortality rate among the three groups (*P* = 0.238).

**Table 2:** Clinical outcomes of the study patients

	Contrast	Air	CO_2_	***P-***values
Technical success	22/22 (100%)	18/18 (100%)	30/30 (100%)	> 0.99
Clinical success	16/22 (72.7%)	13/18 (72.2%)	23/30 (76.6%)	0.472
Number of stents placed				0.697
One stent	7 (31.8%)	3 (16.7%)	5 (16.7%)	
Two stents	10 (45.5%)	11 (61.1%)	19 (63.3%)	
Nasobiliary drain	5 (22.7%)	4 (22.2%)	6 (20%)	
Post-ERCP complications	14 (63.6%)*^#^	5 (27.8%)	8 (26.6%)	0.018
Cholangitis	11 (50%)*^#^	3 (16.7%)	3 (10%)	0.004
Pancreatitis	2 (9.1%)	2 (11.1%)	4 (13.3%)	> 0.99
Bleeding	1 (4.5%)	0	1 (3.3%)	> 0.99
WBC 2 days post ERCP	8.08 ± 2.89	9.48 ± 3.79	7.81 ± 4.17	0.284
Serum bilirubin at one-week post-ERCP (median, [Q1, Q3])	160.39 (99.13, 221.35)	141.98 (86,26, 190.76)	147.55 (81.70, 209.91)	0.817
Mean hospital stay duration at post-ERCP (median, [Q1, Q3])	15.63 (7.89, 19.37)	8.31 (5.08, 11.49)	9.09 (4.37, 12.54)	0.039
ERCP re-intervention during follow-up	1 (4.5%)	1 (5.6%)	3 (10%)	0.854
30-day mortality	4 (18.2%)	2 (11.1%)	1 (3.3%)	0.238

^*^contrast vs. air, ***P*** < 0.05

^#^contrast vs. CO_2_, ***P*** < 0.05

## Discussion

Hilar biliary tumors carry a poor prognosis, with a 5-year overall survival rate of < 10%. Most of these patients are inoperable or unfit for curative resection [17]. Furthermore, palliative surgery is difficult to perform, and is associated with high risk of complications. Endoscopic biliary drainage is the preferred method for palliation. However, post-ERCP cholangitis is the major complication that occurs in 7–57% of patients, according to the different types of obstruction [22, 23]. This risk can reach up to 75% for type IV obstructions [7]. The most common reason for post-ERCP cholangitis is the lack or insufficient drainage of obstructed intrahepatic bile ducts, particularly after contrast injection. Hence, it is important for the endoscopist to avoid excessive contrast injections. MRCP provides useful information on the ductal anatomy, guides endoscopic intervention, and minimizes the use of contrast injections. However, even a small amount of contrast injection without drainage can lead to cholangitis. In a recent study, no significant difference was found between air cholangiography and contrast injection, in terms of the technical and clinical success rates, while a significantly lower incidence of cholangitis was reported with air cholangiography [24]. In the study conducted by De Palma et al., no cholangitis was reported after air cholangiography in patients with infra-hilar obstructions [25]. Sud *et. al.* reported similar results in patients with type II and III hilar obstructions [26]. Although air cholangiogram is effective and decreases the risk of post-ERCP cholangitis, air embolism is a rare and potentially fatal complication. On the other hand, CO_2_ embolism, which is a common event during advanced laparoscopic surgeries, such as laparoscopic hepatectomy due to long operative time and vessel injuries, does not lead to any clinically significant adverse event [27]. Recently, Zhang et al. used CO_2_ as a contrast medium for cholangiography in a small number of patients with Bismuth type II, III and IV lesions. However, the authors did not report any event of CO_2_ embolism in their study, which was likely due to the extremely low volume of CO_2_ injected, when compared to that for direct peroral cholangioscopy [18]. To the best of our knowledge, the present study is the first to demonstrate a significant difference among CO_2_, air and contrast cholangiography in type IV hilar obstructions. In the present study, the incidence of post-ERCP cholangitis for CO_2_, air and the contrast was 10, 16.7 and 50%, respectively (*P <* 0.05). These differences may be explained by the minimal contamination and quicker tissue absorption of air and CO_2_, when compared to the contrast, after injecting this into the biliary system. The hydrostatic law states that the rate of increase in pressure in a vertically downward direction in fluid/liquid is equal to the weight density of the liquid. The density of water at room temperature is taken as 1000 kg/m^3^ (20°C and 1 atm), while that of injected contrast agents is more than that of water. In contrast, the density of air and CO_2_ is 1.205 kg/m^3^ and 1.842 kg/m^3^, respectively, under the same condition. This indicates that when the same volume of contrast, air and CO_2_ is injected into the biliary system, the pressure generated by the latter two media is only < 1/800–1/600 of that generated by the contrast. This significantly lowers the risk of migration of the gut bacteria into the bile duct or blood. In the present study, despite taking all precautionary measures to prevent post-ERCP cholangitis, 17 patients developed cholangitis. At the one-month follow-up, seven patients died from sepsis: one, two and four patients for CO_2_, air and contrast, respectively.

During the endoscopic stenting for malignant hilar obstruction, endoscopists often place the plastic/metal stent in the most dilated bile ducts. Due to various anatomical factors and the level of expertise, no more than two stents are placed in most of these cases. The present study was not designed to determine whether bilateral stenting is better than unilateral stenting, or whether metal stenting is superior to plastic stenting. Merely plastic stents and nasobiliary drains were placed for all cases. It is known that merely 25–30% of the liver volume needs to be drained to improve the clinical and biochemical parameters [28]. Even with MRCP, in some cases, it is difficult to determine whether decompressing the selected bile duct would be adequate, and achieve clinical success. Therefore, temporary nasobiliary drainage was performed to assess the biliary drainage in such cases. If the drainage was clinically successful, the nasobiliary drain was exchanged with plastic stents. In the present study, nasobiliary drainage was utilized for 15 patients: contrast (*n* = 5), air (*n* = 4) and CO_2_ (*n* = 6). Among these 15 patients, five patients (contrast [*n* = 1], air [*n* = 1] and CO_2_ [*n* = 3]) underwent a repeat ERCP, and the nasobiliary drain was changed to stents.

There were some limitations in the present study. First, the sample size was small and the positive rate was low. After consulting statistical experts, the results of multivariate analysis would not be credible. Therefore, we did not conduct multivariate analysis. Second, this was a single-center retrospective study with a short follow-up period. Third, the increasing experience of the endoscopist could have affected the outcomes of the ERCP due to improvement in skill during the study period (from October 2013 to November 2017).

In conclusion, the present study demonstrated that both CO_2_ and air cholangiography are associated with the significantly lower incidence of post-ERCP cholangitis, when compared to contrast injection. However, there was no significant difference in terms of clinical success rate (76.6% vs. 72.2%), post-ERCP cholangitis (10% vs. 16.7%), and reduction in serum bilirubin levels after one week and in the 30-day mortality (3.3% vs. 11.1%) between CO_2_ and air cholangiography. Furthermore, air embolism has been reported during ERCP, and is a known complication. The use of CO_2_ for cholangiography in patients with hilar biliary obstruction during ERCP is recommended.

## Conclusion

In conclusion, ERCP with CO_2_/air cholangiography is feasible and effective for patients with type IV hilar obstructions. Both air and CO_2_ can significantly reduce the risk of cholangitis and hospital stay after ERCP.

## Data Availability

The datasets generated and analysed during the current study are available from the corresponding author on reasonable request.

## Abbreviations

ERCP: Endoscopic retrograde cholangiopancreatography
ALP: Alkaline Phosphatase
γ-GT: Gamma Glutamyl Transpeptidase
WBC: White blood cell
